# Effect of Supercritical Extract from Black Poplar and Basket Willow on the Quality of Natural and Probiotic Drinkable Yogurt

**DOI:** 10.3390/ani11102997

**Published:** 2021-10-19

**Authors:** Marcin Walter, Bartosz Brzozowski, Marek Adamczak

**Affiliations:** Department of Process Engineering, Equipment and Food Biotechnology, Faculty of Food Science, University of Warmia and Mazury in Olsztyn, Jan Heweliusz St. 1, 10-718 Olsztyn, Poland; marcin.walter@uwm.edu.pl (M.W.); marek.adamczak@uwm.edu.pl (M.A.)

**Keywords:** yogurt, supercritical extract, *Populus nigra*, *Salix viminalis*

## Abstract

**Simple Summary:**

Fermented milk products should be an important component of the human diet due to their high nutritional value and easy digestibility. In order to enhance the nutritional value of yogurts, additives in the form of plant-based raw materials are often used. Phytoextracts are a mixture of biologically active compounds that can have beneficial effects on the human body and influence both the manufacturing process and the quality of food products. The incorporation of extracts from the basket willow (*Salix viminalis*) into natural and probiotic yogurts reduced the fermentation time and increased their antioxidant activity. Sensory evaluation of the appearance and texture of yogurts enriched with extracts showed no significant differences compared to natural yogurts. The addition of plant extracts may enable the standardisation of yogurt quality and increase its biological activity.

**Abstract:**

Yogurt is a fermented milk drink produced by *Streptococcus thermophilus*, *Lactobacillus delbrüeckii* ssp. *bulgaricus*, or *Lactobacillus rhamnosus*, which can be enriched with polyphenolic compounds to enhance its antioxidant properties. Supercritical (scCO_2_/H_2_O) extracts obtained from the mixture of bark and wood of black poplar (*Populus nigra*) and basket willow (*Salix viminalis*) are the source of bioactive compounds. The aim of the study was to assess the effect of supercritical extracts from the *P. nigra* and *S. viminalis* on the fermentation, quality, and bioactive properties of drinkable natural and probiotic yogurts. The incorporation of scCO_2_/H_2_O extracts at a dose of 0.01% (w/v) into milk for the production of natural and probiotic yogurts increases their functional properties by enhancing the antioxidant activity without causing negative effects on the physicochemical and organoleptic properties of products. The antioxidant activity of yogurt with scCO_2_/H_2_O extract from *P. nigra* and *S. viminalis* was higher than control yogurts by 1.3–13.2% and 4.4–37.5%, respectively. The addition of a supercritical *S. viminalis* extract reduced the time of natural and probiotic yogurt fermentation. Natural and probiotic yogurt with scCO_2_/H_2_O extracts added was characterised by a bacterial population size of over 7 log cfu/g, and the microflora was active throughout the cold storage period. FTIR analysis confirmed the presence of scCO_2_/H_2_O extracts from *P. nigra* or *S. viminalis* in both types of yogurt. A secondary structure analysis confirmed interactions between compounds of scCO_2_/H_2_O extract from *P. nigra* and *S. viminalis* extract with milk proteins. These interactions affect the compounds’ structural and functional properties by changing, e.g., their digestibility and antioxidant properties.

## 1. Introduction

Yogurt is a fermented milk product that is one of the most common dairy products, a component of everyday diet. It is characterised by a high nutritional value due to its high protein concentration, low-fat concentration or no fat content, and the presence of vitamins and minerals [1]. The consumption of yogurt was demonstrated to have a positive effect on human health, e.g., through increasing muscle mass, lowering LDL cholesterol levels, lowering blood pressure, and protecting against numerous metabolic diseases, i.e., diabetes mellitus and obesity [2]. The lactic acid bacteria found in yogurts have a positive effect on the microbiota of the human gastrointestinal tract, food passage, and immune response [3].

Natural yogurt is obtained by fermenting milk with the participation of lactic acid bacteria belonging to two species: *Streptococcus thermophilus* and *Lactobacillus delbrüeckii* spp. *bulgaricus* [3]. In the production of yogurt, probiotic microorganisms belonging to such species as *L. acidophilus*, *L. rhamnosus*, *L. casei*, *L. fermentum*, *L. reuteri*, *Bifidobacterium longum*, *B. breve*, *B. bifidus,* and *B. infantis* are also used [4]. It is difficult to produce yogurt with a good consistency using only probiotic cultures, without *L. delbrueckii* spp. *bulgaricus* and *S. thermophilus*. Both probiotics and typical yogurt cultures are used as adjunct cultures in probiotic yogurt manufacture [5].

Functional foods are food products that, due to their composition, are characterised by specific nutritional, health-promoting and disease-occurrence reducing properties. Pursuant to Article 2.2 of Regulation 178/2002, as amended, of 2018, issued by the European Parliament and the Council of the European Union, this food should have a protective effect or exhibit therapeutic or medicinal properties [6]. Examples of such foods include natural or probiotic yogurts enriched with fruits, vegetables, or separated polyphenolic compounds, characterised by beneficial antioxidant activity [7,8,9]. Sources of polyphenols include plant raw materials or extracts and fruit juices [10,11,12,13,14,15]. New effective and unconventional extract additives to yogurts are being sought, including extracts from the leaves and seeds of *Moringa oleifera*, the leaves and flowers of *Gnaphalium affine*, the leaves of *Cudrania tricuspidata*, *Morus alba*, the fruit of *Hippophae rhamnoides*, and the bark of *Cinnamomum cassia* [13,16,17,18,19,20].

Unconventional sources of polyphenolic compounds and other biologically active chemical compounds include biomass of perennial industrial plants such as *Miscanthus giganteus*, *M. sachriflorus*, *Spartina pectinata*, *Salix viminalis*, *S. purpurea*, *Populus nigra* × *P. maximowiczii*, *Helianthus salicifolius*, cultivated on marginal land [21]. Marginal land is understood as soils in agricultural use or listed in the agricultural land register, which, due to unfavourable natural, anthropogenic, and economic determinants, are either characterised by low productivity or are not commonly used for food production [21]. According to bioeconomic assumptions, the use of plant biomass for the production of energy carriers is inefficient. It is required to use all plant biomass components, e.g., in the food, feed, pharmaceutical, cosmetic, and chemical industries [22,23].

The *P. nigra*, particularly its buds, leaves, twigs, wood, and bark is a valuable source of bioactive compounds that are extracted using water, organic solvents or supercritical fluids. These extracts contain polyphenols, i.a. cinnamic and benzoic acid derivatives, 5-hydroxy-7-methoxy-flavone, 5,7-dihydroxy-flavone, 5,7-dihydroxy-flavonol, hexanedioic acid, bis(2-ethylhexyl) ester, phthalic acid derivatives, squalene, 3,3,7,11-tetramethyltricyclo [5.4.0.0(4,11)] undecan-1-ol, salicin, populin, gallic acid, saponins, chrysin, and tectochrysin. These compounds exhibit antioxidant, antibacterial, and antifungal properties as well as angiogenic activity, anti-inflammatory, hepatoprotective and vasodilatory-stimulating properties [24,25,26,27,28,29].

Extracts from the leaves, bark, and wood of the *S. viminalis* are composed of fatty acids, aliphatic alcohols, aromatic compounds, terpenoids, tocopherols, sterols, hydroxycinnamic acids and derivatives, benzoic acid derivatives, flavonols, and condensed tannins which exhibit antioxidant, antifungal, antiulcer, bone marrow and blood regenerative after chemotherapy, and *Escherichia coli* inhibitory properties [30,31,32]. In view of the limited information on the applications of extracts from the *P. nigra* and *S. viminalis*, particularly those obtained using supercritical solvents, in the production of food, an attempt was made to determine their effect on the quality of fermented milk products.

The aim of this study was to determine the effect of supercritical extracts from a mixture of the bark and wood of *P. nigra* × *P. maximowiczii* or *S. viminalis* on the quality and antioxidant properties of natural and probiotic yogurts.

## 2. Materials and Methods

### 2.1. Obtaining Extracts from Perennial Industrial Plants

Carbon dioxide extraction was conducted under supercritical conditions from the bark and wood of trees *P. nigra* × *P. maximowiczii* Henry cv. Max–5 and *S. viminalis* L., Ekotur variety, collected in June 2018 from a plantation established on experimental fields of the University of Warmia and Mazury in Olsztyn [21,33]. The extracts were obtained courtesy of the Supercritical Extraction Department, ŁUKASIEWICZ Research Network-New Chemical Syntheses Institute in Puławy, Poland. The extraction was carried out using an industrial installation (Natex, Ternitz, Austria) equipped with two 40 dm^3^ extractors operating at pressures up to 1000 bar and at temperatures up to 90 °C [34]. Shredded tree biomass (approx. 5 kg of each) was extracted using supercritical carbon dioxide with the addition of water (40%, *w*/*w*) (scCO_2_/H_2_O) for nine hours at a temperature of 40 °C and a pressure of 330 bar, using approx. 200 kg CO_2_ per kg of plant material [33]. Water was removed from the obtained extract using a vacuum evaporator at 50 °C (R-220SE, Buchi, Labortechnik AG, Switzerland) and the extract was stored at 5 °C.

### 2.2. Preparation of Drinkable Natural and Probiotic Yogurt

The study used commercially available microorganism cultures for the production of drinkable natural: Yo-Mix 883 (*Streptococcus thermophilus*, *Lactobacillus delbrüeckii* ssp. *bulgaricus*, DuPont, Danisco, France) and probiotic yogurt: Yo-Mix TA460 (*S. thermophilus*, DuPont, Danisco, France), and HOWARU^®^ Rhamnosus (*L. rhamnosus* HN001TM, DuPont, Danisco, France). For the production of yogurt, whole milk powder (Łaciate, Spółdzielnia Mleczarska Mlekpol, Grajewo, Poland) was used. It was dissolved in deionised water heated to 65 °C with the addition of scCO_2_/H_2_O extracts from *P. nigra* and *S. viminalis* (0.01%, *w*/*v*). Milk with extracts and control milk with no extracts were homogenised for 5 min at 24,000 rpm (DI 25 Basic Homogenizer, Staufen, Germany), pasteurised for 10 min at 90 °C, and cooled to 42 °C. Starter cultures were added to milk in the amount indicated in the manufacturer’s instructions. The fermentation of milk was carried out at 42 °C while monitoring the milk acidification dynamics until the curd acidity of pH 4.65 was reached. The yogurt was then cooled to 15 °C, stirred, aseptically poured into sterile 100 cm^3^ bottles and cooled to 5 °C. Yogurt was kept at 5 °C for four weeks, and samples were taken for analyses after 1, 7, 14, 21, and 28 days of storage.

### 2.3. Kinetic Analysis of Milk Acidification

The change in pH value of yogurt samples was measured using a pH-meter (HI 931400, Hanna Instruments, Olsztyn, Poland) during fermentation and refrigerated storage at 5 °C. The acidification kinetics were measured using the changes in pH value of yogurt during fermentation [35]. The maximum acidification rate (V_max_, pH units × 10^−^^3^/min) was calculated from the change in pH value over time (dpH/dt). Kinetic parameters: t_max_ (h), the time taken to reach V_max_, t_pH5.0_ (h) the time taken to reach pH 5.0 and t_f_ (h), the time to complete fermentation, were calculated at the end of fermentation.

The titratable acidity of yogurt samples was determined during cold storage. An ethanolic phenolphthalein solution (0.3 cm^3^, 2%, m/v) was added to yogurt samples (10 g) which were then titrated with an NaOH solution (0.1 mol/dm^3^) to a light pink colour. The titratable acidity was expressed in g of lactic acid per 100 g of yogurt [16]. The analysis was carried out in triplicate.

### 2.4. Analysis of Bacterial Population Size

Decimal dilutions of yogurt samples were prepared in physiological saline. The lactobacillus population size was determined by the submerged culture method. Diluted samples (0.1 cm^3^) were poured into a Petri dish, overlaid by MRS-agar medium (Merck, Darmstadt, Germany) and thoroughly mixed. The incubation was carried out under anaerobic conditions for 48 h, at 37 °C. The streptococcal population size was determined by the method of a surface culture of serially diluted yogurt samples (1 cm^3^) on M17-agar medium (Merck, Darmstadt, Germany). The incubation was carried out under aerobic conditions for 48 h, at 42 °C. The number of colony-forming units (CFUs) on plates containing 30–300 colonies was determined and the number of cells was expressed as log CFU/cm^3^ of yogurts. The analysis was carried out in triplicate.

### 2.5. Determination of Total Polyphenol Concentration (TPC)

Yogurt samples (10 g) were centrifuged (25,000× *g*, 5 °C, 30 min), and the supernatant was filtered (0.45 µm, cellulose acetate) [36]. Total polyphenol concentration (TPC) was determined according to the method by Zhang et al. [37]. Deionised water (0.075 cm^3^), the supernatant (0.025 cm^3^), and Folin–Ciocalteu reagent (0.025 cm^3^, diluted with deionised water at a 1:1 ratio; *v*/*v*) were introduced into the wells of a 96-well microplate. After solution mixing and incubation (6 min, 21 °C), a 7.5% (*w*/*v*) Na_2_CO_3_ solution (0.1 cm^3^) was added to each well. The solution was stirred, the plate was covered with a lid, and the incubation was carried out for 90 min at 21 °C in the absence of light. The absorbance of samples was measured at a wavelength of 765 nm (Multiskan GO, Thermo Scientific, Waltham, MA, USA). The reagent sample was prepared by replacing the actual sample with deionised water. Each sample was analysed in three replications. The polyphenolic concentration was expressed as an equivalent of gallic acid (µg/cm^3^) using a calibration curve within a range of 6.33–202.40 µg/cm^3^ (R^2^ = 0.9981). The analysis was carried out in triplicate.

### 2.6. Determination of Antioxidant Activity

The supernatant obtained after yogurt centrifugation (25,000× *g*, 5 °C, 30 min) was passed through a 0.45 µm porosity filter and used for analyses. The DPPH radical scavenging ability was analysed according to a modified method described by Jung et al. [36]. The supernatant (0.05 cm^3^) and methanolic solution of 1-diphenyl-2-picrylhydrazyl radical (0.15 cm^3^, 0.5 mol/dm ^3^, DPPH; 99.8% methanol, see Supplementary Materials) were introduced into the wells of a 96-well microplate, then stirred and incubated for 30 min at 21 °C. The absorbance of samples was measured at a wavelength of 517 nm (Multiskan GO, Thermo Scientific, Waltham, MA, USA). The determination was conducted in three replications, and the DPPH radical scavenging ability was expressed as an equivalent of Trolox (µg/cm^3^) using a calibration curve within a range of 5.0–50.0 µg/cm^3^ (R^2^ = 0.9976). The analysis was carried out in triplicate.

### 2.7. FTIR Measurements

The spectroscopic measurements were performed by an attenuated total reflectance (ATR) method. The transmittance of yogurt (0.5 cm^3^) samples was measured using an FTIR (IRSpirit-T, Schimadzu, Duisburg, Germany) spectrometer equipped with a QATR-S accessory with a diamond crystal and a DLATGS detector with a germanium-coated KBr beam splitter. Each sample was scanned 25 times within the wavelength range of 4000.0 to 400.0 cm^−1^, at the resolution of 4.0 cm^−1^, using the Happ-Genzel function. Sample spectra obtained with the application of LabSolutions IR software (ver. 2.23, Shimadzu, Duisburg, Germany) were baseline corrected and smoothed with the seven-point Savitzky-Golay algorithm [38]. Furthermore, yogurt spectra deconvolution with Gaussian curve fitting was conducted using Omnic Software (ver. 9.2.86, Thermo Fisher Scientific Inc., Waltham, MA, USA) with the interpretation of the amide I band (1600–1700 cm^−1^) component [39]. The analysis was carried out in triplicate.

### 2.8. Evaluation of Syneresis

The susceptibility of yogurt to syneresis was determined by a method proposed by Robitaille et al. [40]. Yogurt samples (10 g) were gently poured into preweighted 30 cm^3^ polypropylene centrifuge test tubes, centrifuged at 500× *g*, for 10 min at 4 °C. The supernatant whey was gently removed and weighed. Syneresis was calculated as the weight of whey in grams per weight of 100 g of yogurt and expressed in percentage. The analysis was carried out in triplicate.

### 2.9. Analysis of Colour and Sensory Evaluation of Yogurts

Colour assessment was conducted using an instrumental measurement (Chroma Meter CR-400, Konica Minolta) by the CIELAB method. Before the testing, the apparatus was calibrated using a white standard. The C.I.E. system enables the determination of the image brightness (L*), which has values ranging from 0 for black to 100 for white, with the green and red spectra (a*) taking values ranging from −60 (green) to +60 (red), and the blue and yellow spectra (b*) taking values ranging from −60 (blue) to +60 (yellow). Each yogurt sample was stirred, the measurement was carried out six times, and the average colour parameters were determined to be used to calculate the total colour difference (ΔE*) according to the formula [41]:

ΔE* = ((L1* − L2 *) ^2^ + (a1* − a2*) ^2^ + (b1* − b2*) ^2^) ^0.5^ (1)

where L1* was the brightness of yoghurt with scCO_2_/H_2_O, L2* was the brightness of the control yoghurt, a1* was the colour component of yoghurt with scCO_2_/H_2_O, a2* was the colour component of the control yoghurt, b1* was the colour component of yoghurt with scCO_2_/H_2_O, and b2* was the colour component of the control yoghurt.

The analysis of the results was conducted based on the following criterion: between 0 and 1 are absolute colour differences (ΔE*) that are unrecognisable; between 1 and 2, these are slight changes recognisable by a person experienced in recognising colour differences; between 2 and 3.5, this is an average difference recognisable by even an unexperienced person; between 3.5 and 5, this is a distinct difference; above 5, this is a great difference in the colour. The analysis was carried out at least in triplicate.

The scaling method was used in the sensory evaluation of yoghurts. This method is based on the quantitative determination of the quality and sensory intensity of yoghurt in terms of selected characteristics. The sensory attributes, including the appearance, texture, aroma, flavour, colour, and acceptability of yogurt samples important for its quality and played a leading role in the choice of the product by the consumer were selected and were analysed organoleptically by trained panellists. The panellists were people with extensive experience in evaluating standard fermented dairy products such as yoghurt, kefir, and cheese. The panellists underwent initial training in the field of sensory evaluation of selected discriminants with the use of various types of test solutions of flavour and aroma compounds of different consistency and colour, taking into account the specificity of the yoghurts under assessment. Each panellist received three cups of coded yogurt samples. The evaluation was scored on a 10-point scale (0—bad; 10—excellent) according to Anuyahong et al. [35].

### 2.10. HS-SPME-GC-MS Analysis

The headspace solid-phase microextraction/gas-chromatography–mass spectrometry (HS-SPME-GC-MS) technique was used to analyse the volatile compounds of yogurt and yogurt samples supplemented with plant extracts. The analysis was performed based on the optimised method presented by Dan et al. [42]. Briefly, a 5 cm^3^ sample was mixed in a 20 cm^3^ glass vial with PTFE/silicone septum for 5 min at 50 °C to reach equilibrium. An SPME fibre Divinylbenzene/Carboxen™/Polydimethylsiloxane (50/30 µm DVB/Carboxen/PDMS, Supelco, Bellefonte, PA, USA) was exposed in the headspace for 60 min under the same conditions. Volatile compounds were desorbed after the fibre was inserted in the injection port (270 °C, 5 min) of a gas chromatograph GC-2010 Plus (Shimadzu, Duisburg, Germany). An analysis was performed using DB-5ms column (Phenomenex, 30 m, 0.25 mm, 0.25 µm) with helium as the carrier gas at 1 cm^3^/min. The column temperature was initially set at 35 °C for 5 min and then increased to 140 °C at a rate of 4 °C/min for 5 min, heated to 250 °C at a rate of 10 °C/min, and finally held at 250 °C for 5 min. The ion source and transfer line temperatures were 230 and 250 °C, respectively. The mass spectra were recorded using a scan range of 40–400 m/z with the electron impact set at 70 eV. Volatile compounds were identified using a GCMS-TQ4040 mass spectrometer and identified by comparing their mass spectra and retention times with data in the National Institute of Standards and Technology database (NIST 14 mass spectra database). The relative contents of the main volatile compounds were presented. The analysis was carried out in triplicate.

### 2.11. Statistical Analysis

Statistica software (ver. 13.3, TIBCO Software Inc., Palo Alto, CA, USA) was used to perform one-way ANOVA and a Bonferroni test at 95% confidence level (*p* < 0.05) to identify differences among samples. All data presented are mean values followed by the standard deviation.

## 3. Results and Discussion

The previously conducted experiments demonstrated a favourable composition and properties of the extracts obtained using scCO_2_ with an addition of water from a mixture of the bark and wood of *P. nigra* and *S*. *viminalis* [33]. The extracts were characterised by high antioxidant activity and contained phenolic acids, salicylic compounds, and flavonoids in a free form or as esters and glycosides [33]. The extracts from the *P*. *nigra* and *S*. *viminalis* can affect the activity of enzymes and microorganisms, including those used for yogurt production.

### 3.1. Acidification Kinetics and Acidity Measurements during Yogurt Storage

Only the addition of scCO_2_/H_2_O extract from *S*. *viminalis* had an effect on the milk acidification dynamics (Table 1). The addition of this extract resulted in (*p* < 0.05) reduction in the milk acidification rate (V_max_) using probiotic culture from 10.18 ± 0.09 × 10^−3^ to 9.52 ± 0.06 × 10^−3^ pH units/min, and when used for natural yogurt production, it increased (*p* < 0.05) acidification rate from 11.93 ± 0.07 × 10^−3^ to 13.49 ± 0.12 × 10^−3^ pH units/min. Similar relationships were observed for other parameters describing the milk acidification kinetics, i.e., the time (t_max_) in which yogurt cultures reach the maximum values of acidification rate (V_max_), the time (t_pH5.0_) for the yogurt to reach the acidity of pH 5.0, and the total time (t_f_) of milk fermentation. Irrespective of the starter culture, no effect on the milk acidification dynamics was demonstrated with the scCO_2_/H_2_O extract from *P*. *nigra*. The addition of analysed scCO_2_/H_2_O extracts reduced (*p* < 0.05) the duration of milk fermentation by a probiotic culture compared to the control yogurt. 

The addition of plant extracts from green tea, the leaves of *Moringa oleifera*, *Cudrania tricuspidata*, and *Morus alba*, and the seeds of *Salvia hispanica*, was demonstrated to increase the dynamics of milk acidification by yogurt culture bacteria, and to reduce the duration of fermentation [13,19,43,44]. However, Alwazeer et al. [45] report that the addition of extract from *Rheum ribes* increases the duration of milk fermentation by *S*. *thermophilus* and *L. delbrüeckii* ssp. *bulgaricus*, similarly to the addition of extract from grapes [10]. The addition of extract from riceberry rice had no effect on the kinetics of milk acidification with *S*. *thermophilus*, *L*. *delbrüeckii* ssp. *bulgaricus*, *L*. *acidophilus* LA-5, and *B*. *animalis* ssp. *lactis* BB-12 [35].

The active acidity of natural yogurts containing scCO_2_/H_2_O extracts from *P*. *nigra* and *S*. *viminalis*, after one day of cold storage, resulted in pH values of 4.62 ± 0.02 and 4.65 ± 0.01, respectively, and did not differ significantly (*p* < 0.05) from the acidity of yogurt obtained without scCO_2_/H_2_O extracts (Table 2). The pH value of probiotic yogurt containing scCO_2_/H_2_O extracts was significantly higher than that of control yogurt. Irrespective of the starter culture type, over the 28 days of storage, the pH of yogurts with and without the addition of scCO_2_/H_2_O extracts decreased to the values of 4.42–4.48 and 4.52–4.58, respectively. Probiotic and natural yogurts with the addition of scCO_2_/H_2_O extracts were characterised by a significantly (*p* < 0.05) higher pH value throughout the storage period compared to control yogurts. Similarly, the pH value of natural yogurts with scCO_2_/H_2_O extracts was statistically significantly (*p* < 0.05) higher than the control yogurt (Table 2).

The titratable acidity of natural and probiotic yogurts without and with the scCO_2_/H_2_O extracts, increased statistically significantly (*p* < 0.05) during cold storage (Table 2). The titratable acidity value ranged from 0.76 ± 0.01 g lactic acid/100 g at the beginning of storage to 0.97 ± 0.00 g lactic acid/100 g yogurt on day 28 of storage. These parameters meet the criteria specified in the Codex Alimentarius, according to which yogurt acidity should be no less than 0.6 lactic acid in 100 g yogurt [46]. Lactic acid concentration was significantly (*p* < 0.05) lower than that in control yogurt during cold storage of probiotic yogurts with the addition of supercritical extracts. Changes in yogurt acidity during storage are determined, i.a. by its initial acidity, bacterial activity, and cold storage temperature [47,48,49]. The effect of plant extracts and polyphenolic compounds on microorganisms and the milk acidification rate is determined by the extract type, the microbial strains, and the conditions of production and storage [10,36,50,51,52].

### 3.2. Changes in Bacterial Population Size

The survivability of the bacteria used in yogurt technology is influenced by many factors, including the composition and quality of microorganisms, the duration of fermentation, and nutrient availability [43]. On day 1 of storage, in natural yogurt (culture Yo-Mix 883), both control and that with scCO_2_/H_2_O extracts from *P*. *nigra* and *S. viminalis*, the populations of *S*. *thermophilus* and *L*. *delbrüeckii* ssp. *bulgaricus* were 9.25 ± 0.05–9.35 ± 0.04 and 8.30 ± 0.02–8.47 ± 0.06 log cfu/g, respectively (Table 3). After 28 days of storage, the counts of *S*. *thermophilus* and *L*. *delbrüeckii* ssp. *bulgaricus* significantly (*p* < 0.05) decreased to the values of 8.86 ± 0.04–9.00 ± 0.04 log cfu/g and 7.83 ± 0.02–7.93 ± 0.01 log cfu/g, respectively. No significant (*p* < 0.05) effect of supercritical extracts from *P*. *nigra* and *S*. *viminalis* on *S. thermophilus* growth in natural yogurt was found on days 1, 7, and 14 of storage as compared to the control yogurt. On days 21 and 28 of storage, a significantly (*p* < 0.05) greater streptococcal population in control natural yogurt was observed than in yogurts supplemented with analysed scCO_2_/H_2_O extracts from *P*. *nigra* and *S. viminalis*. However, the *L*. *delbrüeckii* ssp. *bulgaricus* population of yogurt supplemented with scCO_2_/H_2_O extract from *S*. *viminalis* was significantly smaller (*p* < 0.05) throughout the storage period. No effect of the scCO_2_/H_2_O extract from *P*. *nigra* on the lactobacilli population size in natural yogurt was demonstrated.

The population of *S. thermophilus* and *L. rhamnosus* in the control probiotic yogurt (cultures Yo-Mix TA460 and HOWARU^®^ Rhamnosus) and probiotic yogurt with scCO2/H2O extracts from *P. nigra* and *S. viminalis*, statistically significantly (*p* < 0.05) decreased after 28 days of cold storage from 8.10 ± 0.01–9.06 ± 0.02 to 7.46 ± 0.02–8.86 ± 0.04 log cfu/g (Table 3). Supercritical extracts from *P. nigra* and *S. viminalis* significantly (*p* < 0.05) contributed to a reduction in the population size of both *S. thermophilus* and *L. rhamnosus* as compared to control probiotic yogurt. 

To have a positive effect on human health, the yogurt microflora population should be at least 7 log cfu/g throughout the storage period [17]. Polyphenols can inhibit the growth of yogurt bacteria, e.g., a high gallic acid concentration disrupts the integrity of the cellular wall of *Lactobacillus* bacteria [50,53]. The addition of a 0.5% extract from red ginseng restricted the development of *L*. *acidophilus* from day 15 of cold storage, while stimulating the development of *S*. *thermophilus* [36]. However, Michael et al. [54] demonstrated (during the storage of yogurt with a mixture of extracts from olives, onions, and citrus fruit) a reduction of viable *S*. *thermophilus* cell counts and an increase in the survivability of *L. delbrüeckii* ssp. *bulgaricus*.

The interactions between polyphenols and proteins or polysaccharides can change the biological activity of polyphenols and thus affect the survivability of microorganisms [50] and have a protective effect on yogurt microflora [55]. Polyphenols from grape extract reduced the activity of a yogurt culture composed of *L*. *acidophilus*, *Bifidobacterium*, *S*. *thermophilus,* and *L*. *delbrüeckii* ssp. *bulgaricus* and significantly increased the survivability of microflora during cold storage for 12 days [10]. Moreover, the extract from *Gnaphalium affine* had no effect on the metabolic activity of *L*. *delbrüeckii* ssp. *bulgaricus* and *S. thermophilus* [17]. Polyphenols and the products released by biotransformation can affect the yogurt microflora. Sun-Waterhouse et al. [51] report that polyphenols from blackcurrant fruit are transformed into more active derivatives, which, under specific conditions, can increase the activity of yogurt strains. The different effects of bioactive extract components on microorganisms results from their different chemical composition and antioxidant activity [10,51,56].

### 3.3. Total Phenolic Content and Antioxidant Activity

The concentration of the compounds reacting with the Folin–Ciocalteu reagent after day 1 of storage in natural and probiotic yogurt was 54.6 ± 2.9 and 46.7 ± 1.1 µg GAE/cm^3^, respectively (Table 4). Control yogurts with no scCO_2_/H_2_O extracts added, both natural and probiotic, exhibited lower antioxidant activity. Antioxidants in yogurts include low molecular weight antioxidants, free amino acids, peptides, and proteins [18]. The level of these compounds in yogurt are affected by the thermal processing of milk, organic acid biosynthesis by lactic acid bacteria and enzymatic hydrolysis of whey proteins and casein, which are a source of bioactive peptides [20].

The addition of supercritical extracts from *P*. *nigra* and *S*. *viminalis* to milk increased (*p* < 0.05) the polyphenolic concentration in yogurts (Table 4). The highest total polyphenolic concentration was noted on day 28 of storage of drinkable yogurt or probiotic yogurt containing scCO_2_/H_2_O extract from *S*. *viminalis* and amounted to 74.2 ± 2.3 and 72.4 ± 4.1 µg GAE/cm^3^, respectively. During storage, the polyphenol concentrations in probiotic yogurt and probiotic yogurt with scCO_2_/H_2_O extract from *P. nigra* varied and at the end of the storage period their concentration was significantly higher than that at the beginning of storage (*p* < 0.05), reaching values of 62.4 ± 4.4 and 71.0 ± 3.0 µg GAE/cm^3^, respectively. Similarly, the total phenolic concentration in probiotic yogurt containing scCO_2_/H_2_O extract from S. *viminalis* significantly (*p* < 0.05) increased during storage from 60.8 ± 1.8 to 74.2 ± 2.3 µg GAE/cm^3^. During storage, in both samples of natural yogurt (the control and with added scCO_2_/H_2_O extracts), the total phenolic concentration significantly (*p* < 0.05) increased from 46.7 ± 1.1−59.9 ± 2.3 µg GAE/cm^3^ on day 1 to 58.0 ± 2.0−72.4 ± 4.1 µg GAE/cm^3^ on day 28. Similar changes in the total phenolic concentration during cold storage of yogurt with the addition of grape pomace and beet extract were presented by Demirkol and Terakci [11] and Flores-Mancha et al. [57], respectively. The addition of polyphenol-containing scCO_2_/H_2_O extracts from *P. nigra* and *S. viminalis* significantly (*p* < 0.05) increased the antioxidant activity of natural and probiotic yogurts (Table 4). Similar to phenolic compound contents, antioxidant activity also significantly (*p* < 0.05) changed during storage. On day 1 of storage, antioxidant activity was 38.0 ± 1.5–56.2 ± 4.7 µg of Trolox/cm^3^, and significantly (*p* < 0.05) increased to 44.3 ± 2.3–57.5 ± 2.3 µg of Trolox/cm^3^. On day 14, the lowest antioxidant activity was noted for all produced yogurts but in the subsequent days, the value of this parameter significantly (*p* < 0.05) increased to reach, on day 28, the maximum values of 56.8 ± 2.2 and 61.6 ± 1.6 µg of Trolox/cm^3^ for probiotic and natural yogurts containing scCO_2_/H_2_O extracts from *S*. *viminalis*, respectively.

The addition of plant extracts obtained from cinnamon, strawberries, blueberry flower pulp, and palm spikelet increased the antioxidant activity of yogurt [12,18,58,59]. The higher antioxidant activity of yogurt with an extract from grapes and grapevine callus was due to the incorporation of phenolic acids such as gallic acid, caffeic acid, coumaric acid, vanillic acid, gentisic acid, vanillin, catechin, epicatechin, trans-resveratrol, hesperidin, and quercetin into the milk [60]. The supercritical extracts from *P*. *nigra* and *S*. *viminalis*, contained polyphenols at concentrations of 24.8 ± 0.7 and 21.2 ± 0.5 µg GAE/cm^3^, respectively, and exhibited antioxidant activity of 49.31 ± 0.8 and 59.6 ± 2.7 µg of Trolox/cm^3^, respectively. The antioxidant activity of yogurts with the addition of scCO_2_/H_2_O extracts was lower than the sum of activities of plain yogurts and scCO_2_/H_2_O extracts from *P*. *nigra* or *S*. *viminalis*. This is probably due to the interactions between polyphenols and milk proteins and other milk components. The measurements of antioxidant activity were conducted for supernatants following the centrifugation of yogurt samples. Therefore, only free, unbound forms of compounds exhibiting antioxidant activity were determined. The release of polyphenolic compounds into the supernatant from natural and probiotic yogurts enriched with supercritical extracts from *P*. *nigra* and *S*. *viminalis* can be limited due to their binding with milk proteins. This can be confirmed by experiments in which samples of yogurt enriched with aqueous cinnamon extract following digestion in vitro presented higher antioxidant activity than that in native samples [18].

Oliveira et al. [8] and Muniandy et al. [7] pointed out that the formation of complexes between proteins and polyphenols is determined, among others, by the amino acid composition of protein, mainly the number of proline residues, the polyphenolic structure, including the number of compounds containing aromatic rings, and the presence of other components such as saccharides and polysaccharides, which also affect these interactions. Polyphenols undergo transformations to varying degrees, which is determined by the enzymatic complex of lactic acid bacteria used as yogurt culture [19]. Polyphenols added to yogurt, e.g., in the form of blackcurrant fruit, undergo biotransformation and during milk fermentation, glycosidic polyphenolic complexes were hydrolysed and aglycones were released [51]. During milk fermentation by lactic acid bacteria, carbon rings can be split and phenolic acids can be either formed or released. These transformations may result in obtaining new active or inactive biological compounds. Glycoside degradation to aglycones usually increases their free radical scavenging activity [51,61]. The polyphenols found in scCO_2_/H_2_O extracts from *P*. *nigra* and *S*. *viminalis* (due to the action of lactic acid bacteria) can undergo transformations while changing the antioxidant properties of yogurts [33].

### 3.4. FTIR Analysis

The supercritical extracts obtained from *P*. *nigra* and *S*. *viminalis* used in this study are composed of salicylates (salicin, saligenin, salicortin), flavonoids (catechin, quercetin, naringenin), and phenolic acids (ferulic acid, sinapic acid, *p*-coumaric acid, syringic acid, protocatechuic acid, *p*-hydroxybenzoic acid, and caffeic acid) [33]. The recorded FTIR spectra of natural and probiotic yogurts with and without scCO_2_/H_2_O extracts from *P*. *nigra* and *S*. *viminalis* were characterised by peaks specific for the vibrations of chemical bonds found in components typical for this type of product. The excitations of chemical bonds were assigned to the corresponding functional groups based on the literature [16,62,63,64,65,66,67].

The FTIR spectra of the yogurts exhibited absorption within the wavelength range corresponding to the vibrations of the bonds found in proteins, lipids and saccharides (Table 5). A broad absorption band within the wavelength range from 3284 to 3272 cm^−1^ (characteristic of O-H and N-H stretching bonds of saccharides, proteins, and polyphenols) was noted for all yogurt types [65,66,67]. This signal is also characteristic of the O-H stretching bonds found in water molecules [16]. Changes in the spectrum intensity and its slight shifts at approx. 3300 cm^−1^ could indicate interactions between polyphenols and proteins, mainly through the formation of hydrogen bonds [68]. All tested yogurts exhibited absorbance values ranging from 2958 to 2852 cm^−1^, corresponding to the vibrations of asymmetric C-H stretching bonds in the functional groups >CH_2_, CH_3_ and symmetric >CH_2_, the C-H bonds in aromatic rings and the O-CH_3_ bonds found in lipids, lignins, saccharides, and esters [62,63,64,65,66,67]. The presence of ester bonds in all tested yogurts was also confirmed by excitation within the wavelength range of 1744 to 1742 cm^−1^ (Table 5), corresponding to esters and carboxylic acids [65,66,67]. However, the range from 1631 to 1537 cm^−1^ is characteristic of the vibrations of the C-N, C=O stretching and deforming bonds in proteins (amide I and II), and non-conjugated cis C=C bonds of alkenyl groups [62,64,67]. Changes in the excitation intensity and wavelength within the amide I and II band result from interactions between polyphenols and casein through the C=O, C-N, and N-H bonds [68].

All yogurts provided excitation at a wavelength of 1454–1451 cm^−1^, which confirmed the presence of symmetric C-O stretching bonds and CH_3_ bending bonds present in the functional groups of proteins and lipids [65,66,67]. The vibrations of C-O, P=O, >PO_2_, C-O-C, and aromatic C-O stretching bonds within the wavelength range of 1245–1160 cm^−1^, found in esters, phospholipids, and saccharides, were present in all yogurt samples [16,62,63,64,65,66,67]. Natural and probiotic yogurts without scCO_2_/H_2_O extracts and probiotic yogurt with scCO_2_/H_2_O extract from *P. nigra* exhibited a minor excitation within a range of 1096–1072 cm^−1^ (Table 5), resulting from the presence of C-O and C-N stretching bonds and P=O stretching bonds in >PO_2_ in aliphatic amines, esters, phospholipids, glycoproteins, and saccharides [62,64,65,66,67]. The source of excitation at wavelengths of 1054, 1037, 1038, and 1017 cm^−1^ is the addition of scCO_2_/H_2_O extracts from *P*. *nigra* or *S*. *viminalis* to both types of yogurt. At these wavelengths, absorbance is exhibited by C-C bonds, C-H stretching and aromatic bonds, and C-O and C-N stretching bonds found in phenols, pectins, polysaccharides, and aliphatic amines [62,63,64].

Vibrations of the C-O and C-N stretching bonds within a range from 1029 to 1033 cm^−1^ confirm the presence of aliphatic amines in yogurt samples. All samples were characterised by excitation (526–646 cm^−1^) originating from α-glycosidic bonds and C-H bending bonds present in saccharides and isoprenoids [62,63].

When polyphenols bind with proteins, they affect their structural and functional properties by changing their digestibility and antioxidant properties [68]. The secondary structure of proteins that describes the arrangement of polypeptides in the space, resulting from interactions of amino acid functional groups, includes α-helix, β-sheet, β-turns, and random coils [69]. Table 6 provides changes in the percentage proportion of secondary structures in proteins of natural and probiotic yogurt with or without the addition of scCO_2_/H_2_O extracts from *P*. *nigra* or *S*. *viminalis* during cold storage. The dominant protein structure in control natural and probiotic yogurts is β-sheet at a concentration of 39.2 ± 0.0 and 39.5 ± 0.1%, respectively. During the storage of control probiotic yogurt until day 14, the proportion of this structure in protein increases significantly (*p* < 0.05) to a value of 40.0 ± 0.1%, which is followed by a decrease to a value of 32.9 ± 0.1%. However, in control natural yogurt during the storage, the proportion of β-sheet in the protein structure decreased gradually and significantly (*p* < 0.05) to a value of 33.8 ± 0.1% (Table 6).

The addition of supercritical extracts from *P*. *nigra* and *S*. *viminalis* to probiotic yogurt resulted in a significant (*p* < 0.05) decrease in the proportion of β-sheet during cold storage, but this process was less dynamic than that in control samples. However, in natural yogurt with scCO_2_/H_2_O extracts from *P*. *nigra* and *S*. *viminalis*, the proportion of β-sheet varied from 34.2 ± 0.0 to 40.1 ± 0.2%.

Another abundant protein structure type is α-helix at a concentration of 29.4 ± 0.0 and 28.8 ± 0.3% in control natural and probiotic yogurt samples, respectively (Table 6). During storage, its proportion in protein significantly (*p* < 0.05) decreased to a value of 21.5 ± 0.1 and 22.3 ± 0.1% for control natural and probiotic yogurt, respectively. Probiotic yogurt with scCO_2_/H_2_O extract from *P*. *nigra* was characterised by a similar content of α-helix in protein to that in control probiotic yogurt. The addition of scCO_2_/H_2_O extract from *S*. *viminalis* to this yogurt from day 14 of the storage significantly (*p* < 0.05) decreased the proportion of this structure. In natural yogurt with supercritical extracts from *P*. *nigra* and *S. viminalis*, the proportion of α-helix in protein varied during cold storage.

Polyphenols exhibit a strong affinity for globular proteins, causing them to develop their secondary structure [70]. β-sheet is mainly found in folded protein regions, and it is mostly the aggregation of β-lactoglobulin and α-lactalbumin that is responsible for increasing its proportion in the secondary structure [71]. β-sheet can be converted into β-turns, and then into the structure of random coils [72]. Polyphenols added to cow’s milk also interact with casein, decreasing the proportion of α-helix and β-sheet while increasing the proportion of β-turns and random coils. Catechin can form insoluble complexes with β-casein. The affinity of polyphenols for proteins is determined by the molecule size and increases with an increase in the molecular weight of phenolic compounds [70]. The open spatial structure of native casein results from electrostatic, hydrophobic interactions between polypeptide chains, and is determined by the presence of the residues of proline which, when present in polypeptide chains, interferes with the formation of α-helix and β-sheet, thus promoting the formation of turns and hydrophobic regions [70,73]. Polyphenols exhibit such high affinity for proline residue that they can distort and disrupt the structure of large loops and β-sheet. The released polypeptides can be rearranged due to hydrogen interactions to form new fragments which partially take on the structure of α-helix and β-sheet [73].

During cold storage of natural and probiotic yogurts containing scCO_2_/H_2_O extracts from *P*. *nigra* and *S*. *viminalis*, the proportion of intermolecular β-sheet structure of proteins increased significantly (*p* < 0.05). In proteins from probiotic yogurt with scCO_2_/H_2_O extracts from *P. nigra* and *S*. *viminalis*, the proportion of intermolecular β-sheet increased from 13.6 ± 0.1–13.9 ± 0.0% on day 1 to 17.5 ± 0.1–19.1 ± 0.0% on day 28 of storage. Similar changes were noted for natural yogurt with both supercritical extracts (Table 6). On the first day of storage, the proportion of β-turns in the protein structure of control natural yogurt was 14.3 ± 0.1%, and was significantly (*p* < 0.05) greater than that in the samples with the addition of supercritical extracts from *P. nigra* or *S. viminalis* (13.3 ± 0.2 and 13.7 ± 0.2%). From day 7 to 28, yogurts with scCO_2_/H_2_O extract from *S*. *viminalis* were characterised by a greater proportion of β-turns than those of control yogurt. However, polyphenols from the scCO_2_/H_2_O extract from *P*. *nigra* caused a significant increase in the number of β-turns only on days 7 and 14 of natural yogurt storage. The addition of supercritical extracts from *P. nigra* and *S. viminalis* to probiotic yogurt significantly (*p* < 0.05) increased the proportion of β-turns in the protein structure on days 1 and 7 and gradually decreased until day 28 of storage.

In general, the proportion of the random coil structure in protein increased during the storage of natural and probiotic yogurt. On day 1 of storage, the proportion of the random coil structure amounted to 4.3 ± 0.6, 3.1 ± 0.1, and 2.9 ± 0.1%, respectively, for probiotic yogurt, control yogurt, and yogurt with supercritical extracts from *P*. *nigra* and *S. viminalis*. An analysis of the effect of these extracts on the protein structure of this yogurt on individual days revealed a significant (*p* < 0.05) reduction in the proportion of random coils on days 1, 7, and 21 and their increase on days 14 and 28. The greatest proportion of this structure in protein (ranging from 13.3 ± 0.0 to 13.9 ± 0.1%) was noted on the final day of storage. In the experiment with natural yogurt, after day 1 of storage, the initial addition of scCO_2_/H_2_O extracts from *P*. *nigra* and *S. viminalis* significantly (*p* < 0.05) increased the proportion of random coils in the protein structure compared to the control sample. From day 7 to 28, the proportion of random coils in the natural yogurt protein was significantly (*p* < 0.05) lower than in the control yogurt.

Lactic acid bacteria including *S*. *themophilus*, *L*. *delbrüeckii* spp. *bulgaricus*, and *L*. *rhamnosus*, synthesise i.a. glycosidases, peptidases, and esterases involved in enzymatic transformations of lactose, proteins, and milk fat [74]. From the polyphenols incorporated into yogurt, free phenolic acids, flavonoids, and salicylates can be released, which, by interacting with proteins, change their spatial structure. This was confirmed by a study by Lee et al. [75], who demonstrated the ability of probiotics to metabolise polyphenols and release phenolic acids and flavonoids with altered biological activities. A change in the ratio between the secondary protein structures may also result from the action of peptidases and the release of polypeptide fragments which free phenolic acids or flavonoids can bind with. The metabolism of polyphenols by the yogurt culture microflora during milk fermentation includes the hydrolysis of glycoside flavonoid derivatives, the splitting of flavonoid aromatic rings, or the release of compounds with a simpler structure and lower molecular weight from polyphenols [51].

### 3.5. Evaluation of Syneresis

Syneresis is considered one of the most important parameters indicating the quality of yogurt during storage that determines the balance between the attractive forces and the repulsive forces of proteins and the rearrangement of bonds between the casein network components. The protein network structure in yogurts is stabilised by non-covalent interactions (mainly hydrophobic interactions) between amino acid side chains. Polyphenols added to milk can interact with the hydrophobic surface of proteins by reducing the interactions between amino acid hydrophobic side chains. A smaller number of available hydrophobic functional groups can change the protein gel matrix, thus increasing the amount of whey released [10]. In the presented experiments, the addition of scCO_2_/H_2_O extracts from *P. nigra* and *S. viminalis* statistically significantly (*p* < 0.05) increased syneresis in both natural and probiotic yogurts (Table 7). The addition of supercritical extract from *S*. *viminalis* to both yogurt types resulted in the release of serum from day 7 to 28 of storage being significantly greater (*p* < 0.05) than the addition of supercritical extract from *P*. *nigra* (Table 7). The syneresis of natural yogurt supplemented with scCO_2_/H_2_O extracts from *P*. *nigra* and *S. viminalis* ranged from 12.4 ± 0.8 to 13.4 ± 0.8% after one day of storage. During the storage of natural yogurt with scCO_2_/H_2_O extracts from *P*. *nigra* and *S*. *viminalis*, the amount of released whey increased significantly (*p* < 0.05) to reach values ranging from 18.6 ± 0.5 to 19.4 ± 0.0% after 28 days. Similar relationships were obtained in experiments with probiotic yogurt (Table 7).

The increased whey release can also result from the higher titratable acidity of the yogurt obtained with the addition of polyphenols and higher proteolysis due to the increase in milk fermentation duration [76]. The polyphenols found in scCO_2_/H_2_O extracts from *P*. *nigra* and *S*. *viminalis* can rearrange hydrophobic bonds by increasing the pores in the yogurt gel structure, while releasing greater amounts of whey. In turn, the addition of a 0.125–0.5% extract from riceberry rice or blueberry flower pulp to yogurt significantly reduced syneresis [35,58]. The reduction in the amount of released whey is explained by the interactions between polyphenols and casein being stronger than those between polyphenols and other milk proteins, which results in the formation of soluble complexes with a well-developed structure. The spatial structure of the gel, changed in this way, has an increased water-binding capacity [8,35,77].

### 3.6. Organoleptic Evaluation of Yogurts

The effect of supercritical extracts from *P. nigra* and *S*. *viminalis* on the organoleptic properties of natural and probiotic yogurt is presented in Table 8. The assessors found no significant changes in the range of overall acceptability of both types of yogurt with scCO_2_/H_2_O extracts compared to the control samples. No effect of the scCO_2_/H_2_O extracts on the appearance and texture of yogurts was noted (Table 8). However, the results indicate that the scCO_2_/H_2_O extracts to yogurt resulted in a significant (*p* < 0.05) reduction in the rating of their flavour and aroma. The lower rating of these distinguishing features was due to the bitter, astringent flavour and woody aroma of the scCO_2_/H_2_O extracts. The extracts from riceberry rice, horseradish tree, and red ginseng significantly diminished the flavour and aroma of yogurts [13,35,36]. However, the incorporation of extract from grapes or grapevine callus did not significantly change the flavour or aroma of the product [10,60].

In this study, a significant (*p* < 0.05) change in the colour as compared to control samples was only noted for natural and probiotic yogurt with a supercritical extract from *S*. *viminalis*. The difference in colour between control natural and probiotic yogurts and yogurts with an scCO_2_/H_2_O extract from *S*. *viminalis* was ΔE* = 1.01 and ΔE* = 0.96, respectively (Table 9).

Yogurt is recognised as an excellent delivery matrix for plant extracts and phenolic compounds. However, these additives cause quality changes in the product depending on the technology, process parameters, and starter culture composition. Yoghurt supplemented with polyphenols showed changed gastrointestinal digestion and could influence the viability of the yogurt bacteria [78]. Polyphenols influence bacterial activity and could reduce [45] or increase this activity [7] and thus influence yogurt quality attributes, e.g., texture, rheology, and volatile compound profile [77]. More than 90 flavour compounds have been identified so far in yogurt and it has been reported that the aroma and taste of yogurt are mainly due to the presence of non-volatile or volatile acids and carbonyl compounds [79]. The composition of the volatile compounds in yogurt and other fermented milk products depends on the composition of lactic acid bacteria [80]. The most important aromatic components are acetaldehyde, acetone, acetoin, diacetyl and 2,3-butanedione. In the obtained yogurts, the main compounds were 2,3-butanedione (diacetyl, buttery odour), 2,3-pentanedione (a buttery, cheesy, sweet, nutty, fruity odour), and 2-heptanone (a banana-like, fruity odour) (Table 10).

The main compounds detected in plant extract were *n*-hexane, hexanal (oily-green odour), (*E*)-2-heptenal (green type odour), *n*-nonanal (rose-orange odour), and isophorone (peppermint-like odour) (Table 10). The lower rating of flavour and aroma of yogurt supplemented with extracts was due to the bitter, astringent flavour and woody aroma of the scCO_2_/H_2_O extracts. However, the main volatile compounds detected in extracts were not identified in yogurt samples. It could be that the low rate of flavour and aroma was influenced by non-volatile compounds present in plant extracts. Minor compounds could also influence the flavour and aroma evaluation of yogurt. In yogurt supplemented with plant extract, octanoic acid and 1-(1-methylethoxy)propane were detected and these compounds were not present in plant extracts.

## 4. Conclusions

Extracts obtained using scCO_2_/H_2_O from the *P. nigra* and *S. viminalis* can be used as ingredients of natural and probiotic yogurts which contain lactic acid bacterial strains, e.g., *S. thermophilus*, *L. delbrüeckii* ssp. *bulgaricus*, or *L. rhamnosus*. The extract from *S. viminalis* reduced the time of natural yogurt fermentation. The bacterial population size in yogurts with the scCO_2_/H_2_O extracts was no less than 7 log cfu/g and the microflora was active throughout the cold storage period. The incorporation of scCO_2_/H_2_O extracts at a dose of 0.01% (*w*/*v*) into milk for the production of natural and probiotic yogurts increased their functional properties by enhancing antioxidant activity without causing major negative effects on the physicochemical and organoleptic properties of the products. The addition of scCO_2_/H_2_O extracts to both types of yogurt increased syneresis, but the sensory evaluation of the appearance and texture revealed no significant differences compared to natural yogurts.

## Figures and Tables

**Table 1:** Acidification kinetic parameters: V_max_—the maximum acidification rate; t_max_—the time taken to reach V_max_; t_pH5.0_—the time taken to reach pH 5.0; t_f_—the time to complete fermentation, of probiotic (Yp) and natural (Yn) yogurt with or without scCO_2_/H_2_O extracts from *P. nigra* (Pn) and *S. viminalis* (Sv) during fermentation *.

Yogurt Type	Kinetic Parameters			
	V_max_ (10^−3^ pH Units/min)	t_max_ (h)	t_pH5.0_ (h)	t_f_ (h)
Yp	10.18 ± 0.09 aC	1.95 ± 0.02 bB	3.80 ± 0.02 bB	5.78 ± 0.02 bB
Yp+Pn	10.15 ± 0.02 aC	1.97 ± 0.02 abB	3.83 ± 0.02 bB	5.73 ± 0.02 cC
Yp+Sv	9.52 ± 0.06 bD	2.00 ± 0.02 aA	3.90 ± 0.02 aA	5.85 ± 0.02 aA
Yn	11.93 ± 0.07 *b*B	1.88 ± 0.02 *a*C	3.47 ± 0.02 *a*C	5.23 ± 0.02 *a*D
Yn+Pn	11.93 ± 0.07 *b*B	1.88 ± 0.02 *a*C	3.50 ± 0.02 *a*C	5.23 ± 0.02 *a*D
Yn+Sv	13.49 ± 0.12 *a*A	1.72 ± 0.02 *b*D	2.95 ± 0.02 *b*D	4.70 ± 0.02 *b*E

* Mean values of three different determinations followed by standard deviation are presented. a–c, Different letters in a column for probiotic yogurt represent statistical differences (ANOVA, Bonferroni test, *p* < 0.05); *a–b*, Different letters in a column for natural yogurt represent statistical differences (ANOVA, Bonferroni test, *p* < 0.05); A–E, Different letters in a column for yogurt type represent statistical differences (ANOVA, Bonferroni test, *p* < 0.05).

**Table 2:** Changes in the pH value and titratable acidity of probiotic (Yp) and natural (Yn) yogurt with or without scCO_2_/H_2_O extracts from *P. nigra* (Pn) and *S. viminalis* (Sv) during storage *.

Yogurt Type	Days of Storage at 5 °C				
	1	7	14	21	28
pH value					
Yp	4.64 ± 0.02 *a*A	4.61 ± 0.02 *a*A	4.57 ± 0.01 *a*B	4.54 ± 0.01 *a*BC	4.52 ± 0.01 *a*C
Yp+Pn	4.67 ± 0.01 *b*A	4.63 ± 0.01 *a*B	4.61 ± 0.01 *b*BC	4.59 ± 0.02 *b*C	4.58 ± 0.01 *b*C
Yp+Sv	4.67 ± 0.01 *b*A	4.62 ± 0.01 *a*B	4.61 ± 0.01 *b*BC	4.60 ± 0.01 *b*C	4.58 ± 0.01 *b*D
Yn	4.62 ± 0.01 aA	4.49 ± 0.01 aB	4.46 ± 0.01 aC	4.43 ± 0.01 aCD	4.42 ± 0.01 aD
Yn+Pn	4.62 ± 0.02 aA	4.53 ± 0.01 bB	4.49 ± 0.01 bC	4.48 ± 0.01 bC	4.47 ± 0.01 bC
Yn+Sv	4.65 ± 0.01 aA	4.56 ± 0.01 cB	4.51 ± 0.01 cC	4.49 ± 0.01 bD	4.48 ± 0.01 bD
Titratable acidity (g of lactic acid/100 g of yogurt)					
Yp	0.82 ± 0.02 *a*A	0.87 ± 0.00 *a*B	0.92 ± 0.01 *a*C	0.94 ± 0.00 *a*D	0.95 ± 0.00 *a*D
Yp+Pn	0.76 ± 0.01 *b*A	0.84 ± 0.01 *b*B	0.86 ± 0.00 *b*B	0.88 ± 0.01 *b*C	0.91 ± 0.01 *b*D
Yp+Sv	0.76 ± 0.02 *b*A	0.85 ± 0.01 *b*B	0.87 ± 0.01 *b*B	0.90 ± 0.00 *b*C	0.92 ± 0.01 *b*C
Yn	0.85 ± 0.01 aA	0.94 ± 0.01 aB	0.95 ± 0.00 aC	0.96 ± 0.00 aCD	0.97 ± 0.00 aD
Yn+Pn	0.82 ± 0.01 bA	0.92 ± 0.00 bB	0.94 ± 0.00 abC	0.95 ± 0.00 bC	0.96 ± 0.00 aD
Yn+Sv	0.84 ± 0.00 aA	0.92 ± 0.00 bB	0.93 ± 0.01 bC	0.95 ± 0.01 abD	0.96 ± 0.01 aD

* Mean values of three different determinations followed by standard deviation are presented. a–c, Different letters in a column for probiotic yogurt represent statistical differences (ANOVA, Bonferroni test, *p* < 0.05); *a–b*, Different letters in a column for natural yogurt represent statistical differences (ANOVA, Bonferroni test, *p* < 0.05)*;* A–D, Different letters in a row for days of storage represent statistical differences (ANOVA, Bonferroni test, *p* < 0.05).

**Table 3:** Viable counts (log cfu/g) of *S. thermophilus* (S.th.), *L. rhamnosus* (L.rh.), and *L. bulgaricus* (L.b.) in probiotic (Yp) and natural (Yn) yogurt with or without scCO_2_/H_2_O extracts from *P. nigra* (Pn) and *S. viminalis* (Sv) during storage *.

Yogurt Type	Bacteria Strain	Days of Storage at 5 °C				
		1	7	14	21	28
Yp	S.th.	9.06 ± 0.02 *a*A	9.04 ± 0.02 *a*A	8.97 ± 0.05 *a*AB	8.90 ± 0.05 *a*B	8.86 ± 0.04 *a*B
Yp+Pn		8.91 ± 0.02 *b*A	8.73 ± 0.04 *b*B	8.58 ± 0.06 *b*C	8.22 ± 0.05 *b*D	8.18 ± 0.03 *b*D
Yp+Sv		8.83 ± 0.06 *b*A	8.64 ± 0.04 *b*B	8.41 ± 0.01 *c*C	8.19 ± 0.02 *b*D	8.12 ± 0.07 *b*D
Yp	L.rh.	8.41 ± 0.07 aA	8.18 ± 0.02 aB	8.13 ± 0.06 aB	8.09 ± 0.02 aBC	7.99 ± 0.03 aC
Yp+Pn		8.13 ± 0.04 bA	7.87 ± 0.06 bB	7.81 ± 0.04 bBC	7.76 ± 0.03 bBC	7.70 ± 0.03 bC
Yp+Sv		8.10 ± 0.01 bA	7.70 ± 0.05 cB	7.61 ± 0.07 cB	7.61 ± 0.03 cB	7.46 ± 0.02 cC
Yn	S.th.	9.35 ± 0.04 *a*A	9.34 ± 0.04 *a*A	9.22 ± 0.06 *a*AB	9.16 ± 0.06 *a*B	9.00 ± 0.04 *a*C
Yn+Pn		9.27 ± 0.04 *a*A	9.22 ± 0.01 *a*AB	9.16 ± 0.02 *a*B	9.01 ± 0.01 *b*C	8.89 ± 0.04 *b*D
Yn+Sv		9.25 ± 0.05 *a*A	9.24 ± 0.06 *a*A	9.17 ± 0.06 *a*A	9.00 ± 0.04 *b*B	8.86 ± 0.04 *b*B
Yn	L.b.	8.47 ± 0.06 aA	8.32 ± 0.05 aB	8.27 ± 0.05 aBC	8.18 ± 0.04 aC	7.93 ± 0.01 aD
Yn+Pn		8.37 ± 0.01 abA	8.28 ± 0.02 aB	8.17 ± 0.03 abC	8.04 ± 0.02 bD	7.88 ± 0.04 abE
Yn+Sv		8.30 ± 0.02 bA	8.26 ± 0.01 aA	8.14 ± 0.03 bB	7.96 ± 0.04 bC	7.83 ± 0.02 bD

* Mean values of three different determinations followed by standard deviation are presented. *a–c*, Different letters in a column for *S. thermophilus* represent statistical differences (ANOVA, Bonferroni test, *p* < 0.05)*;* a–c, Different letters in a column for *L. rhamnosus* or *L. bulgaricus* represent statistical differences (ANOVA, Bonferroni test, *p* < 0.05)*;* A–E, Different letters in a row for days of storage represent statistical differences (ANOVA, Bonferroni test, *p* < 0.05).

**Table 4:** Total phenolic content and antioxidant activity of probiotic (Yp) and natural (Yn) yogurt with or without scCO_2_/H_2_O extracts from *P. nigra* (Pn) and *S. viminalis* (Sv) during storage *.

Yogurt Type	Days of Storage at 5 °C				
	1	7	14	21	28
Total phenolic content (µg of GAE/cm^3^)					
Yp	54.6 ± 2.9 *a*AB	54.9 ± 1.5 *a*AB	49.0 ± 1.1 *a*A	54.3 ± 3.0 *a*AB	62.4 ± 4.4 *a*B
Yp+Pn	60.1 ± 1.6 *b*A	65.9 ± 2.3 *b*AB	59.9 ± 1.9 *b*A	61.5 ± 1.1 *b*A	71.0 ± 3.0 *ab*B
Yp+Sv	67.7 ± 3.1 *b*AB	63.3 ± 4.0 *b*A	60.8 ± 1.8 *b*A	66.4 ± 1.6 *b*AB	74.2 ± 2.3 *b*B
Yn	46.7 ± 1.1 aA	49.1 ± 1.1 aA	58.5 ± 5.6 aB	54.8 ± 2.1 aAB	58.0 ± 2.0 aB
Yn+Pn	58.6 ± 2.9 bAB	56.5 ± 1.6 bA	64.1 ± 3.7 aB	62.9 ± 0.7 bAB	63.7 ± 1.9 aB
Yn+Sv	59.9 ± 2.3 bA	61.7 ± 3.8 bA	60.8 ± 2.7 aA	67.5 ± 1.0 cAB	72.4 ± 4.1 bB
Antioxidant activity (µg of Trolox/cm^3^)					
Yp	38.0 ± 1.5 *a*AB	44.3 ± 2.3 *a*B	33.2 ± 0.9 *a*A	39.2 ± 2.0 *a*AB	43.9 ± 1.2 *a*B
Yp+Pn	40.0 ± 3.4 *ab*A	46.2 ± 3.2 *a*B	37.5 ± 2.4 *a*A	44.1 ± 1.7 *b*B	47.7 ± 0.8 *b*B
Yp+Sv	47.1 ± 4.2 *b*AB	49.9 ± 2.0 *a*ABC	43.8 ± 1.6 *b*A	53.6 ± 1.2 *c*BC	56.8 ± 2.2 *c*C
Yn	46.5 ± 4.1 aB	48.4 ± 1.8 aB	39.1 ± 0.6 aA	42.7 ± 1.8 aAB	45.6 ± 2.7 aAB
Yn+Pn	47.1 ± 0.4 abABC	51.3 ± 1.5 aBC	43.5 ± 1.6 abA	46.9 ± 2.0 aAB	51.6 ± 1.9 bC
Yn+Sv	56.2 ± 4.7 bB	57.5 ± 2.3 bB	46.7 ± 3.1 bA	58.8 ± 1.6 bB	61.8 ± 1.6 cB

* Mean values of three different determinations followed by standard deviation are presented. *a–c*, Different letters in a column for probiotic yogurt represent statistical differences (ANOVA, Bonferroni test, *p* < 0.05)*;* a–c, Different letters in a column for natural yogurt represent statistical differences (ANOVA, Bonferroni test, *p* < 0.05)*;* A–C, Different letters in a row for days of storage represent statistical differences (ANOVA, Bonferroni test, *p* < 0.05).

**Table 5:** Wavenumbers of QATR-FTIR peaks and functional groups of natural (Yn) and probiotic (Yp) yogurt with or without scCO_2_/H_2_O extracts from *P. nigra* (Pn) and *S. viminalis* (Sv).

Wavenumbers (cm^−1^)						Band Assignment	Component
Yn	Yn+ Pn	Yn+ Sv	Yp	Yp+ Pn	Yp+ Sv		
3276	3275	3276	3272	3278	3284	OH and N-H stretching	carbohydrates, proteins, polyphenolic
2956	2958	2958	2957	2957	2957	C-H asymmetric stretching in CH_3_ and >CH_2_, C-H symmetric stretching in >CH_2_, C-H aromatic stretching, O-CH_3_	lipids, lignin, saccharides, and esters
2921	2923	2923	2921	2922	2923		
2852	2853	2853	2852	2852	2853		
1742	1743	1743	1743	1743	1744	C=O stretching in -O-(C=O)-O	saturated esters or carboxylic acid
1632	1632	1632	1632	1632	1632	C=O, C-N stretching, non-conjugated C=C	amide I band proteins
1537	1537	1537	1537	1537	1537	N-C reformatting	amide II band proteins
1454	1454	1454	1452	1452	1451	C-O symmetrical stretching, CH_3_ bending	lipids, proteins
1243	1243	1243	1243	1243	1245	C-O stretching in -(O=)C-O-, P=O stretching in >PO_2_	esters, phospholipids
1163	1161	1162	1169	1162	1160	C-O-C stretching, C-O ring vibrations	carbohydrates
1093	-	-	1096	-	-	C-O stretching; C-N stretching	aliphatic amines
1080	-	-	1072	1080	-	C-O stretching, P=O stretching in >PO_2_	esters, phospholipids, carbohydrates, glycoprotein
-	1054	1054	-	-	-	C-H aromatic, stretching; C-O and C-N stretching	phenolic, aliphatic amines
-	-	-	-	1038	1037	C-O stretching, C-N stretching	aliphatic amines
-	1033	1033	1033	1028	1029	C-O stretching, C-N stretching	aliphatic amines
-	1017	1017	-	-	-	C-O, C-C and C-N stretching	polysaccharides, pectins
646	601	549	617	567	526	alpha-glycosidic bond, C-H bending	carbohydrates, isoprenoids

**Table 6:** Secondary structures of amide I band (QATR-FTIR) from probiotic (Yp) and natural (Yn) yogurt with or without scCO_2_/H_2_O extracts from *P. nigra* (Pn) and *S. viminalis* (Sv) during storage *.

Days of Storage at 5 °C	Yogurt Type	Secondary Structure (%)				
		β-Sheet	Intermolecular β-Sheet	β-Turn	α-Helix	Random Coil
1	Yp	39.5 ± 0.1 bB	13.6 ± 0.1 bI	13.8 ± 0.2 bAB	28.8 ± 0.3 bB	4.3 ± 0.6 aK
	Yp+Pn	39.2 ± 0.1 bBC	13.9 ± 0.1 aI	14.2 ± 0.1 aA	29.6 ± 0.1 aA	3.1 ± 0.1 bL
	Yp+Sv	39.2 ± 0.1 aBCD	13.8 ± 0.0 aI	14.2 ± 0.1 aA	29.9 ± 0.1 aA	2.9 ± 0.1 bL
7	Yp	39.5 ± 0.3 aB	15.8 ± 0.2 aH	11.0 ± 0.5 cF	21.5 ± 0.2 abDE	12.2 ± 0.3 aEF
	Yp+Pn	39.0 ± 0.0 bCD	16.3 ± 0.3 aG	12.0 ± 0.1 bE	21.3 ± 0.1 bE	11.5 ± 0.1 bG
	Yp+Sv	38.8 ± 0.1 bD	15.9 ± 0.1 aGH	13.1 ± 0.1 aC	21.8 ± 0.0 aD	10.5 ± 0.2 cI
14	Yp	40.0 ± 0.1 aA	17.2 ± 0.3 aEF	12.4 ± 0.1 bDE	20.9 ± 0.1 aF	9.6 ± 0.1 cJ
	Yp+Pn	38.0 ± 0.1 bE	17.0 ± 0.1 aF	11.7 ± 0.2 cE	20.9 ± 0.1 aF	12.5 ± 0.0 aDE
	Yp+Sv	38.0 ± 0.1 bE	17.0 ± 0.1 aF	12.8 ± 0.1 aCD	20.8 ± 0.1 aFG	11.4 ± 0.0 bGH
21	Yp	35.8 ± 0.1 cG	18.0 ± 0.0 aBC	12.8 ± 0.1 aCD	20.6 ± 0.0 aFG	12.8 ± 0.0 aCD
	Yp+Pn	37.8 ± 0.2 aE	17.7 ± 0.2 abCD	13.0 ± 0.3 aCD	20.7 ± 0.1 aFG	10.8 ± 0.0 cHI
	Yp+Sv	36.8 ± 0.1 bF	17.6 ± 0.1 bCDE	13.3 ± 0.2 aBC	20.6 ± 0.0 aFG	11.8 ± 0.0 bFG
28	Yp	32.9 ± 0.1 cI	17.5 ± 0.1 cDE	14.0 ± 0.2 aA	22.3 ± 0.1 aC	13.3 ± 0.0 cBC
	Yp+Pn	35.1 ± 0.2 bH	19.1 ± 0.0 aA	11.4 ± 0.2 bEF	20.5 ± 0.1 bG	13.9 ± 0.1 aA
	Yp+Sv	37.2 ± 0.2 aF	18.2 ± 0.1 bB	11.5 ± 0.1 bEF	19.4 ± 0.1 cH	13.7 ± 0.0 bAB
1	Yn	39.2 ± 0.0 aAB	13.9 ± 0.1 aI	14.3 ± 0.1 aAB	29.4 ± 0.0 aA	3.3 ± 0.1 cH
	Yn+Pn	38.6 ± 0.1 bBC	12.9 ± 0.2 bJ	13.3 ± 0.2 cC	26.7 ± 0.5 cC	8.5 ± 0.9 aF
	Yn+Sv	38.8 ± 0.1 bBC	13.1 ± 0.1 bJ	13.7 ± 0.2 bBC	28.0 ± 0.4 bB	6.4 ± 0.8 bG
7	Yn	35.7 ± 0.7 aEFG	15.5 ± 0.5 bH	12.3 ± 0.2 bEF	23.9 ± 0.3 bE	12.6 ± 0.3 aC
	Yn+Pn	35.0 ± 0.1 aFGH	16.0 ± 0.3 abFGH	13.1 ± 0.1 aCDE	24.8 ± 0.1 aD	11.0 ± 0.6 bDE
	Yn+Sv	34.7 ± 0.3 aGHI	16.7 ± 0.1 aDEF	13.4 ± 0.3 aBC	25.4 ± 0.4 aD	9.7 ± 0.5 cEF
14	Yn	36.3 ± 0.3 cDE	15.8 ± 0.4 aGH	10.9 ± 0.4 bGI	20.2 ± 0.7 aH	16.8 ± 0.2 aA
	Yn+Pn	40.1 ± 0.2 aA	16.0 ± 0.3 aGH	11.9 ± 0.3 aFG	21.4 ± 0.1 aFG	10.7 ± 0.4 cDE
	Yn+Sv	38.2 ± 0.1 bC	16.5 ± 0.0 aEFG	12.4 ± 0.2 aDEF	21.1 ± 0.1 abFG	11.9 ± 0.2 bCD
21	Yn	35.2 ± 0.8 cFG	17.7 ± 0.3 aBC	11.0 ± 0.6 bGHI	20.6 ± 0.2 aGH	15.5 ± 0.7 aAB
	Yn+Pn	38.2 ± 0.2 aC	17.3 ± 0.1 abCD	11.9 ± 0.1 bFG	20.7 ± 0.1 aGH	12.0 ± 0.0 bCD
	Yn+Sv	36.9 ± 0.1 bD	17.2 ± 0.1 bCDE	13.2 ± 0.1 aCD	20.8 ± 0.1 aGH	12.0 ± 0.0 bCD
28	Yn	33.8 ± 0.1 cI	18.8 ± 0.1 aA	11.4 ± 0.4 bI	20.5 ± 0.1 bG	15.6 ± 0.3 aAB
	Yn+Pn	34.2 ± 0.0 bHI	18.2 ± 0.1 bAB	11.3 ± 0.0 bGH	20.9 ± 0.1 aGH	15.4 ± 0.0 bB
	Yn+Sv	35.7 ± 0.2 aEF	16.1 ± 0.1 cFGH	14.9 ± 0.1 aA	20.6 ± 0.2 cG	12.7 ± 0.1 cC

* Mean values of three different determinations followed by standard deviation are presented. a–c, Different letters in a column for yoghurt type on different days of storage represent statistical differences in a day (ANOVA, Bonferroni test, *p* < 0.05)*;* A–L, Different letters in a column for yogurt type represent statistical differences in all storage periods (ANOVA, Bonferroni test, *p* < 0.05).

**Table 7:** Changes in the syneresis (%) of probiotic (Yp) and natural (Yn) yogurt with or without scCO_2_/H_2_O extracts from *P. nigra* (Pn) and *S. viminalis* (Sv) during storage *.

Yogurt Type	Days of Storage at 5 °C				
	1	7	14	21	28
Yp	25.2 ± 0.2 *a*A	29.9 ± 0.9 *a*B	30.3 ± 1.0 *a*B	30.7 ± 0.2 *a*B	30.9 ± 0.9 *a*B
Yp+Pn	26.3 ± 0.5 *b*A	32.8 ± 0.0 *b*B	33.0 ± 0.5 *b*B	33.3 ± 0.2 *b*BC	33.7 ± 0.2 *b*C
Yp+Sv	25.1 ± 0.1 *b*A	36.1 ± 0.9 *c*B	37.2 ± 0.4 *c*C	37.7 ± 0.2 *c*C	38.9 ± 0.0 *c*D
Yn	9.6 ± 0.4 aA	93.1 ± 0.3 aA	11.7 ± 0.5 aB	14.7 ± 0.5 aC	15.2 ± 0.1 aC
Yn+Pn	12.4 ± 0.8 bA	15.0 ± 0.5 bB	16.1 ± 0.1 bB	17.7 ± 0.3 bC	18.6 ± 0.5 bC
Yn+Sv	13.4 ± 0.8 bA	17.9 ± 0.9 cB	18.6 ± 0.4 cBC	19.0 ± 0.0 cBC	19.4 ± 0.0 cC

* Mean values of three different determinations followed by standard deviation are presented. a–c, Different letters in a column for probiotic yogurt represent statistical differences (ANOVA, Bonferroni test, *p* < 0.05)*;* a–c, Different letters in a column for natural yogurt represent statistical differences (ANOVA, Bonferroni test, *p* < 0.05)*;* A–D, Different letters in a row for days of storage represent statistical differences (ANOVA, Bonferroni test, *p* < 0.05).

**Table 8:** Sensory evaluation of probiotic (Yp) and natural (Yn) yogurt with or without scCO_2_/H_2_O extracts from *P. nigra* (Pn) and *S. viminalis* (Sv) on the first day of storage *.

Yogurt Type	Sensory Characteristics (Points)					
	Appearance	Texture	Aroma	Flavour	Colour	Acceptability
Yp	9.9 ± 0.2 *a*	9.8 ± 0.4 *a*	9.0 ± 0.6 *a*	7.8 ± 0.2 *a*	8.9 ± 0.6 *a*	7.0 ± 0.6 *a*
Yp+Pn	9.9 ± 0.2 *a*	10.0 ± 0.1 *a*	7.5 ± 0.4 *b*	6.4 ± 0.6 *b*	8.7 ± 0.5 *ab*	6.8 ± 0.6 *a*
Yp+Sv	9.9 ± 0.2 *a*	9.8 ± 0.4 *a*	7.5 ± 0.2 *b*	6.0 ± 0.6 *b*	8.0 ± 0.6 *b*	6.4 ± 0.5 *a*
Yn	10.0 ± 0.0 a	10.0 ± 0.1 a	7.8 ± 0.6 a	7.1 ± 0.3 a	9.0 ± 0.3 a	7.5 ± 0.6 a
Yn+Pn	10.0 ± 0.0 a	10.0 ± 0.1 a	5.5 ± 0.5 b	6.0 ± 0.2 b	8.8 ± 0.2 a	6.7 ± 0.8 a
Yn+Sv	10.0 ± 0.1 a	10.0 ± 0.1 a	4.9 ± 0.4 b	5.8 ± 0.5 b	8.2 ± 0.4 b	6.5 ± 0.7 a

* Mean values of three different determinations followed by standard deviation are presented. *a–c*, Different letters in a column for probiotic yogurt represent statistical differences (ANOVA, Bonferroni test, *p* < 0.05); a–c, Different letters in a column for natural yogurt represent statistical differences (ANOVA, Bonferroni test, *p* < 0.05).

**Table 9:** The colour characteristic of probiotic (Yp) and natural (Yn) yogurt with or without scCO_2_/H_2_O extracts from *P. nigra* (Pn) and *S. viminalis* (Sv) on the first day of storage *.

Yogurt Type	Colour Characteristic			ΔE*
	L*	a*	b*	
Yp	88.30 ± 0.18 *b*	−3.34 ± 0.03 *b*	9.31 ± 0.05 *a*	-
Yp+Pn	88.42 ± 0.09 *b*	−3.24 ± 0.02 *a*	9.36 ± 0.02 *a*	0.17
Yp+Sv	87.56 ± 0.10 *a*	−3.43 ± 0.03 *c*	9.91 ± 0.05 *b*	0.96
Yn	89.53 ± 0.20 c	−3.31 ± 0.02 b	8.83 ± 0.03 a	-
Yn+Pn	88.93 ± 0.08 b	−3.19 ± 0.01 a	9.03 ± 0.02 b	0.65
Yn+Sv	88.64 ± 0.16 a	−3.38 ± 0.01 c	9.32 ± 0.02 b	1.01

* Mean values of three different determinations followed by standard deviation are presented. *a–c*, Different letters in a column for probiotic yogurt represent statistical differences in each colour measurement (ANOVA, Bonferroni test, *p* < 0.05); a–c, Different letters in a column for natural yogurt represent statistical differences in each colour measurement (ANOVA, Bonferroni test, *p* < 0.05); L*, darkness-lightness (0–100); a*, greenness-redness (−60 ± 60). b*, blueness-yellowness (−60 ± 60); ΔE*, total colour difference.

**Table 10:** The main volatile compounds detected in probiotic (Yp) and natural (Yn) yogurt with or without scCO_2_/H_2_O extracts from *P. nigra* (Pn) and *S. viminalis* (Sv).

Volatile Compounds	RT (min)	Relative Concentration (%)							
		Pn	Sv	Yp	Yp+Pn	Yp+Sv	Yn	Yn+Pn	Yn+Sv
Acetalaldehyde	0.61	nd *	nd	2.08	0.18	1.28	nd	nd	nd
Ethanol	1.44	nd	nd	0.25	nd	0.24	nd	0.73	0.14
2,3-Butanedione	2.16	nd	nd	16.23	17.51	19.46	51.46	56.43	51.79
*n*-Hexane	2.27	14.39	9.85	nd	nd	nd	nd	nd	nd
Acetic acid	2.36	0.57	nd	nd	nd	nd	nd	nd	nd
Ethyl acetate	2.47	nd	0.67	nd	1.59	nd	nd	1.59	nd
2-Pentanone	3.67	nd	nd	2.70	3.49	3.47	1.94	1.74	1.15
2,3-Pentanedione	3.86	nd	nd	8.59	11.57	10.79	3.00	2.45	2.40
*n*-Heptane	3.96	10.99	5.91	0.52	nd	nd	nd	nd	nd
1-Pentanol	6.33	nd	nd	2.47	3.19	2.11	nd	2.66	1.59
Hexanal	7.60	3.22	4.83	0.84	1.53	nd	nd	nd	nd
1-Hexanol	10.82	nd	nd	2.33	2.69	1.87	nd	2.01	1.09
2-Heptanone	11.72	nd	nd	13.03	13.21	9.72	8.47	5.65	3.39
Methyl caproate	13.37	nd	0.54	nd	nd	nd	nd	nd	nd
(*E*)-2-Heptenal	14.73	8.71	4.09	nd	nd	nd	nd	nd	nd
1-Octen-3-one	15.75	3.12	1.50	nd	nd	nd	nd	nd	nd
1-Octen-3-ol	15.84	1.10	0.75	nd	nd	nd	nd	nd	nd
Octanal	16.85	1.05	1.06	nd	nd	nd	nd	nd	nd
2-Nananone	20.65	nd	nd	2.14	2.77	1.46	nd	nd	0.47
Linalool	20.98	0.97	nd	nd	nd	nd	nd	nd	nd
*n*-Nonanal	21.17	2.17	3.23	nd	nd	nd	nd	nd	nd
Isophorone	21.78	2.97	2.75	nd	nd	nd	nd	nd	nd
Methyl caprylate	22.02	1.89	2.22	nd	nd	nd	nd	nd	nd
α-Terpineol	24.65	0.51	0.70	nd	nd	nd	nd	nd	nd
Decenal	25.22	1.02	1.95	nd	nd	nd	nd	nd	nd
Methyl nonanoate	25.94	1.75	2.20	nd	nd	nd	nd	nd	nd
(*E*)-2-Decenal	27.32	1.45	1.47	nd	nd	nd	nd	nd	nd
*n*-Tridecan-1-ol	34.04	nd	0.79	nd	nd	nd	nd	nd	nd

* nd—not detected.

## Data Availability

Data sharing not applicable.

## Supplementary Materials

The following are available online at https://www.mdpi.com/article/10.3390/ani11102997/s1, Chemical reagents were used in the experiments.
