# Supplementary material for: Effect of Supercritical Extract from Black Poplar and Basket Willow on the Quality of Natural and Probiotic Drinkable Yogurt
Source: Animals (Basel). 2021 Oct 19;11(10):2997. doi: 10.3390/ani11102997 (PMC8532730; doi:10.3390/ani11102997)
Supplement: Supplementary file 1 [file animals-11-02997-s001.zip › animals-1395370-SI.pdf]

Effect of supercritical extract from black poplar and basket willow on the quality of natural and probiotic drinkable yogurt

SUPPLEMENTAL MATERIAL

Marcin Walter [1], Bartosz Brzozowski [1,*] and Marek Adamczak [1]

[1] Department of Process Engineering, Equipment and Food Biotechnology, Faculty of Food Science, University of Warmia and Mazury in Olsztyn, Jan Heweliusz St. 1, 10-718 Olsztyn, Poland; bartosz.brzozowski@uwm.edu.pl (B.B); marek.adamczak@uwm.edu.pl (M.A.); marcin.walter@uwm.edu.pl (M.W.)

* Correspondence: bartosz.brzozowski@uwm.edu.pl; Phone: +48-89523-36-50 (B.B.)

Chemicals

The following chemical reagents were used in the experiments: methanol (≥99%, Stanlab, Lublin, Poland), Folin–Ciocalteu reagent (Aktyn, Suchy Las, Poland), sodium carbonate (≥99%, Stanlab), sodium hydroxide (≥99%, Stanlab), sodium chloride (≥99%, Stanlab), gallic acid (≥98%, Sigma Aldrich, Poznan, Poland), 2,2-diphenyl-1-picrylhydrazyl, (DPPH, Sigma Aldrich), quercetin (≥95% Sigma Aldrich) and 6-hydroxy-2,5,7,8-tetramethylchromane-2-carboxylic acid (Trolox, ≥98%, Sigma Aldrich).
